# Resting-state brain plasticity is associated with the severity in cervical spondylotic myelopathy

**DOI:** 10.1186/s12891-024-07539-2

**Published:** 2024-06-06

**Authors:** Yongming Tan, Ziwei Shao, Kaifu Wu, Fuqing Zhou, Laichang He

**Affiliations:** 1 Department of Radiology, First affiliated hospital, Jiangxi Medical College, Nanchang University, Nanchang, 330006 China; 2 Clinical Research Center for Medical Imaging of Jiangxi Province, Nanchang, Jiangxi Province China; 3 Department of Radiology, Wuhan Central Hospital, Wuhan, China

**Keywords:** Cervical spondylotic myelopathy, Resting-state functional magnetic resonance imaging, Degree centrality, Brain plasticity

## Abstract

**Objective:**

To investigate the brain mechanism of non-correspondence between imaging presentations and clinical symptoms in cervical spondylotic myelopathy (CSM) patients and to test the utility of brain imaging biomarkers for predicting prognosis of CSM.

**Methods:**

Forty patients with CSM (22 mild-moderate CSM, 18 severe CSM) and 25 healthy controls (HCs) were recruited for rs-fMRI and cervical spinal cord diffusion tensor imaging (DTI) scans. DTI at the spinal cord (level C2/3) with fractional anisotropy (FA) and degree centrality (DC) were recorded. Then one-way analysis of covariance (ANCOVA) was conducted to detect the group differences in the DC and FA values across the three groups. Pearson correlation analysis was then separately performed between JOA with FA and DC.

**Results:**

Among them, degree centrality value of left middle temporal gyrus exhibited a progressive increase in CSM groups compared with HCs, the DC value in severe CSM group was higher compared with mild-moderate CSM group. (*P* < 0.05), and the DC values of the right superior temporal gyrus and precuneus showed a decrease after increase. Among them, DC values in the area of precuneus in severe CSM group were significantly lower than those in mild-moderate CSM and HCs. (*P* < 0.05). The fractional anisotropy (FA) values of the level C2/3 showed a progressive decrease in different clinical stages, that severe CSM group was the lowest, significantly lower than those in mild-moderate CSM and HCs (*P* < 0.05). There was negative correlation between DC value of left middle temporal gyrus and JOA scores (*P* < 0.001), and the FA values of dorsal column in the level C2/3 positively correlated with the JOA scores (*P* < 0.001).

**Conclusion:**

Structural and functional changes have taken place in the cervical spinal cord and brain of CSM patients. The Brain reorganization plays an important role in maintaining the symptoms and signs of CSM, aberrant DC values in the left middle temporal gyrus may be the possible mechanism of inconsistency between imaging findings and clinical symptoms. Degree centrality is a potentially useful prognostic functional biomarker in cervical spondylotic myelopathy.

Cervical spondylotic myelopathy (CSM) is the compression of the spinal cord or spinal cord ischemia due to the degeneration of the cervical intervertebral disc and its adjacent tissues [1]. Recently, some functional magnetic resonance imaging (fMRI) studies have demonstrated that there is structural and functional plasticity in patients with cervical spondylotic myelopathy at resting state in the brain [2–6]. However, it is unclear if the reorganization occurring in these CSM patients is associated with the severity of spinal cord compression and if severity of spinal cord compression influences functional recovery.

Degree centrality(DC) is a powerful fMRI tool to explore the communication potential strength of given nodes or reveal the core hub architecture of whole brain. As we all known, the human brain is a highly complex network system consisting of interactions of neurons and neuronal clusters [7]. Voxel-wise DC explores the properties of the whole-brain functional connectome at the voxel level and is the most direct analysis method to describe the influence and function of network nodes [8]. Unlike seed-based functional connectivity [9] it does not require a priori assumptions and can directly assess the connectivity patterns of whole brain functional networks, thus tapping into the physiological basis of the brain’s intrinsic functional framework.

The diagnosis of CSM requires an agreement between symptoms of myelopathy and MR imaging findings of spinal cord compression in T2 imaging. Diffusion tensor imaging (DTI) has high sensitivity in assessing subtle lesions in the nervous system and can detect spinal cord lesions before T2 hyperintensities appear [10, 11]. FA has been reported by several groups to correlate with CSM severity and may represent an adjunct marker to help guide surgical intervention [12, 13]. . However, it found that the severity of cervical spinal cord compression was not a simple one-to-one correspondence with clinical symptoms [14, 15]. It is common in clinical practice that some CSM patients have mild herniated intervertebral discs and severe clinical symptoms, while others have severe herniated disc but lighter clinical manifestations. The bizzare phenomenon means asymptomatic spondytic cervical cord compression [16]or imaging of cervical cord compression not well-matched with clinical status [17], in which the specific mechanism behind it is worth exploring.

The aim of this study intends to combine DC of brain and the characteristics of cervical spinal cord DTI changes to investigate the possible mechanisms of inconsistency between imaging presentations and clinical symptoms in patients with CSM, and to provide imaging evidence for the objective assessment of early CSM disease. We hypothesized that cortical and subcortical reorganization would be greater in patients with more severe spinal injury, and that patients with severe injury would have more impaired neurological function despite increased DC measured cortical activity.

## Methods

### Clinical data

The study was approved by the Ethics Committee of the First Affiliated Hospital of Nanchang University (2014-037), and written informed consent was obtained from each subject before the study.

We calculated the sample size using this G-power software. We set effect size, α err prob, power and number of groups to 0.4, 0.05, 0.8 and 3 respectively, the total sample size is then calculated. A total of forty right-handed patients with clinically diagnosed CSM (20 males, 20 females, mean age: 48.98 ± 6.24 years) from the First Affiliated Hospital of Nanchang University, and were enrolled in this study from October 2016 to December 2019. Two radiologists independently examined the radiological features of each patient. Thereafter, a diagnosis of CSM was determined by a clinician and the two radiologists. Besides, patients should meet these following inclusions: [1] volunteer to enroll in the study; [2] clear evidence of cord compression on a cervical spine MRI, such as an ossified posterior longitudinal ligament, herniated discs, and demyelination with hyperintensity of the cord on T2WI; [3] no medication therapy or decompression surgery; and [4] were right-handed. The CSM group met the pertinent exclusion criteria: (1) psychiatric disorders or major neurologic disorders (e.g., epilepsy, severe stroke or visible lesions); (2) history of neck surgery before; (3) age < 30 or > 65; (4) contraindications to MRI. All patients underwent clinical protocol including the Japanese Orthopaedic Association Scores (JOA) (maximum score 17) [12]and Neck Disabilitv Index (NDI) questionnaires. The JOA score system evaluates disease severity and sensorimotor function, and the NDI was designed to measure the activities of daily living in patients with neck pain. CSM was divided into two groups: mild-moderate group (17 ≥ JOA ≥ 9), and severe group (JOA < 9). Meanwhile, we also recruited 25 healthy controls matched in age, sex, and education, and all were right-handed. None of healthy controls had any history of neurological or psychiatric diseases.

### MRI data acquisition

All MRI datasets were obtained using a 3.0T MR scanner (Trio; Siemens, Erlangen, Germany) equipped with a 16-channel radio‐frequency receive head and neck coil. These datasets included fMRI dataset and DTI images. During the functional scans, the participants were instructed to relax with their eyes closed but not to fall asleep. The datasets of rs-fMRI were acquired using an echo-planar imaging (EPI) sequence with the following parameters: repetition time (TR) = 2000ms, echo time (TE) = 30ms, flip angle (FA) = 90 °, field of view (FOV) = 200 × 200 mm, matrix = 64 × 64, voxel size = 3.0 × 3.0 × 4.0 mm^3^, slice number = 30, slice gap = 1.2 mm, slice thickness = 4 mm. Total of 240 time points (8 min, 6 s). Diffusion tensor imaging covering the cervical spinal cord was acquired using spin-echo single-shot EPI sequence: TR/TE = 5000/100ms; FOV = 109 × 109 mm; NEX = 2; matrix = 128 × 124; slice thickness = 7 mm; 20 non-collinear diffusion-weighted gradient directions with b = 600 s/mm^2^ and one additional image without diffusion weighting (i.e., b = 0 s/mm^2^). All participants underwent routine MRI examination of the head and neck before resting-state scans to exclude obvious lesions.

### MRI data processing (Fig. 1)

**Fig. 1:**
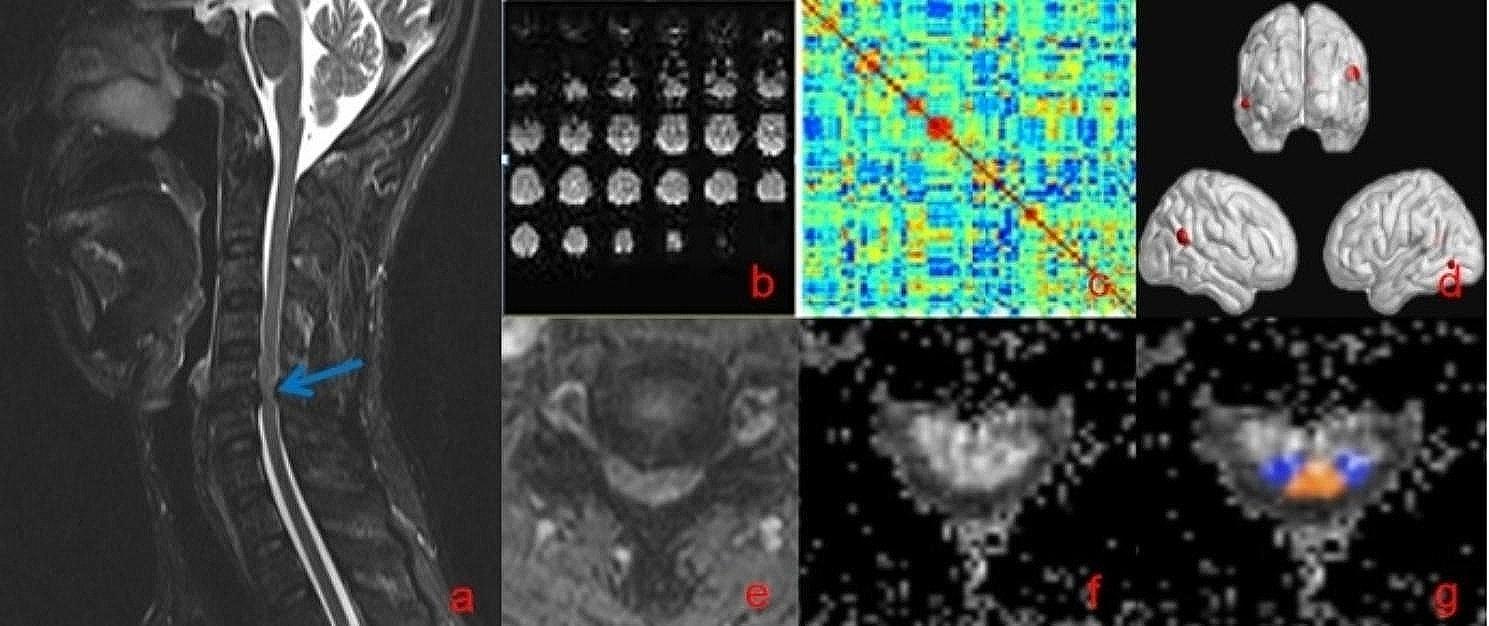
A flowchart of data processing. **(a)** sagittal T2WI MR image of a patient with CSM; **(b)** rs-fMRI data; **(c)**: weighted connectivity matrix; **(d)** DC differences; **(e)** transverse T2WI MR image; **(f)** FA of the proximal spinal cord; **(g)** probabilistic maps of the lateral corticospinal tracts (blue) and dorsal columns (yellow) following registration to the SCT atlas

#### Brain fMRI data processing

Preprocessing of raw data was performed with Data Processing & Analysis for Brain Imaging (DPABI 2.1, http://www.rest-fmri.net) toolbox which was based on MATLAB R2013b (Mathworks, Natick, MA, USA). Data processing included removing the first 10 time points, slice timing correction, head motion correction (the participants who had more than 2.0 mm or 2.0◦ head motion were rejected), normalization into Montreal Neurological Institute (MNI) space and resliced by 3.0 × 3.0 × 3.0mm^3^ voxels, removing linear trends, filtering (0.01 ~ 0.08 Hz) and nuisance covariate regression (including white matter, CSF and the head movement parameters). After these preprocessing steps, fMRI data were used for DC calculations. We used weighted DC, because it provides a more precise centrality characterization of functional brain networks than binary methods. To obtain each subject’s graph, we transformed the Pearson’s correlation data into normally distributed Fisher Z-scores and constructed the whole-brain functional network by thresholding each correlation at *r* > 0.25, then a weighted DC value was obtained for each voxel. Because spatial smoothing can introduce artificial local correlations, we performed smoothing with a Gaussian kernel of 6 mm FWHM [18] after DC calculations, as described in previous studies [19].

### Cervical spine data processing

DTI datasets were analyzed by using the Spinal Cord Toolbox [20], Version4.0 (SCT; https://www.nitrc.org/projects/sct/). Exclude images with low signal or artifacts and the main steps are described below [21]: (1) compute mean diffusivity map from the datasets; (2) segment spinal cord. Segmentation errors can be resolved by automatic segmentation or manual editing; (3) motion correction; (4) register template to diffusivity map; (5) compute diffusion tensor. Metrics are calculated with outlier rejection by using the RESTORE (robust estimation of tensors by outlier rejection) metho.

### Statistics

To detect differences in the demographic and clinical data, we compared age, sex, years of education, JOA score, NDI score among the three groups by conducting one-way analysis of variance (ANOVA) and chi-square (χ^2^) test was used for categorical data. The statistical significance was set at P value less than 0.05. For voxel-wise degree centrality, we first reported within-group statistic map of DC measurement for CSM groups and HCs group using one-sample t-test (*P* < 0.05, FDR corrected). Then one-way analysis of covariance (ANCOVA) was conducted to detect the group differences in the DC maps across the three groups with age, sex, and years of education as covariates(voxel *P* < 0.001, cluster *P* < 0.05, FDR corrected). A Bonferroni correction was used for multiple comparisons in post-hoc analyses reporting formal tests of significance. Finally, to determine whether abnormal DC or FA values is associated with clinical characteristics (including JOA and NDI) of CSM, we conducted a Pearson correlation analysis in patients.

## Results

### Demographics and clinical characteristics

The demographics and clinical data of the participants in this study are shown in Table 1. No significant differences were observed in age (F = 0.935, *P* = 0.398), sex (χ^2^ = 0.429, *P* = 0.807), years of education (F = 0.743, *P* = 0.480) between CSMs and the HCs. The mean JOA and NDI levels for CSM patients (mild-moderate and severe groups) were 11.950 ± 1.889, 6.830 ± 1.150, 32.32%±6.77% and 48.28%±8.41%, respectively.

**Table 1:** Demographic and clinical characteristics of the subjects

	Mild-Moderate CSM	Severe CSM	HCs	*P*-value
Age (year)	48.091 ± 6.872	50.056 ± 5.363	47.320 ± 7.022	0.398^c^
Sex (M/F)	12/10	8/10	13/12	0.807^d^
Education (year)	11.227 ± 2.224	10.500 ± 1.855	11.200 ± 2.179	0.480
JOA	11.950 ± 1.889 ^a^	6.830 ± 1.150 ^a^	-	-
NDI	32.32%±6.77% ^a^	48.28%±8.41% ^a^	-	-
Motor function	6 (4, 6)^b^	4 (3, 5)^b^	-	-
Upper limb movement	3 (2, 3)^b^	3 (1, 3)^b^	-	-
Lower limb movement	3 (2, 3)^b^	2 (1, 3)^b^	-	-
Sensory function	4 (3, 4)^b^	3 (2, 5)^b^	-	-
Upper limb sensation	1 (1)^b^	1 (0,1)^b^	-	-
Lower limb sensation	1 (1, 2)^b^	1 (1, 2)^b^	-	-
Trunk sensation	2 (1, 2)^b^	1(0,2)^b^	-	-

*Abbreviations* CSM-cervical spondylotic myelopathy; HC-healthy control; NDI-Neck Disability Index; JOA-Japanese Orthopedic Association

a-the measurement data conforming to normal distribution, expressed as mean ± standard deviation

b-the measurement data that did not conform to normal distribution, expressed as median (interquartile spacing)

c-the *p* value obtained by ANOVA

d-the *p* value obtained by χ^2^-test

### DTI parameters of spinal white matter fiber tracts at C2-3 level of the subjects

**Fig. 2:**
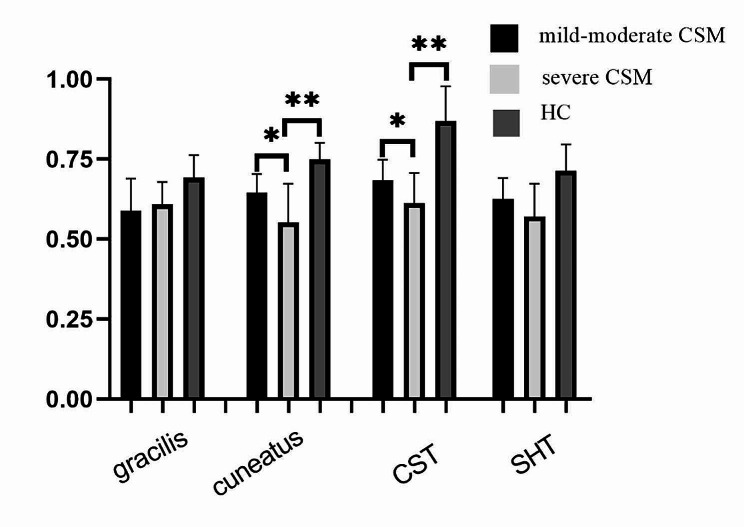
Differences of FA in spinal white matter fiber tracts at C2-3 level of the subjects. *Abbreviations* CST-corticospinal tract; SHT-spinothalamic tract; **-*p* < 0.01; *p < 0.05

The FA values of cuneatus and corticospinal tract in the level C2/3 showed a progressive decrease in different clinical stages, that severe CSM group was the lowest, significantly lower than those in mild-moderate CSM and HCs (*P* < 0.05)(Table 2; Fig. 2.

**Table 2:** Differences of FA in spinal white matter fiber tracts at C2-3 level of the subjects

	Mild-Moderate CSM	Severe CSM	HCs	F	*P*-value
L-gracilis	0.749 ± 0.058	0.719 ± 0.053	0.788 ± 0.154	12.346	0.016
R-gracilis	0.743 ± 0.072	0.710 ± 0.058	0.862 ± 0.065	10.322	0.024
L-cuneatus	0.708 ± 0.052	0.664 ± 0.060	0.844 ± 0.090	9.369	0.030
R-cuneatus	0.711 ± 0.081	0.670 ± 0.046	0.832 ± 0.080	11.483	0.000
L-lateral corticospinal tract	0.713 ± 0.079	0.664 ± 0.076	0.952 ± 0.080	15.752	0.000
R-lateral corticospinal tract	0.713 ± 0.075	0.678 ± 0.064	0.946 ± 0.078	16.789	0.000
L-spinothalamic tract	0.757 ± 0.057	0.695 ± 0.062	0.831 ± 0.085	10.986	0.000
R-spinothalamic tract	0.742 ± 0.091	0.706 ± 0.073	0.828 ± 0.088	6.879	0.000

Note There are no obvious difference between left and right sides of gracilis, cuneatus, lateral corticospinal tract and spinothalamic tract

### DC differences among groups

**Fig. 3:**
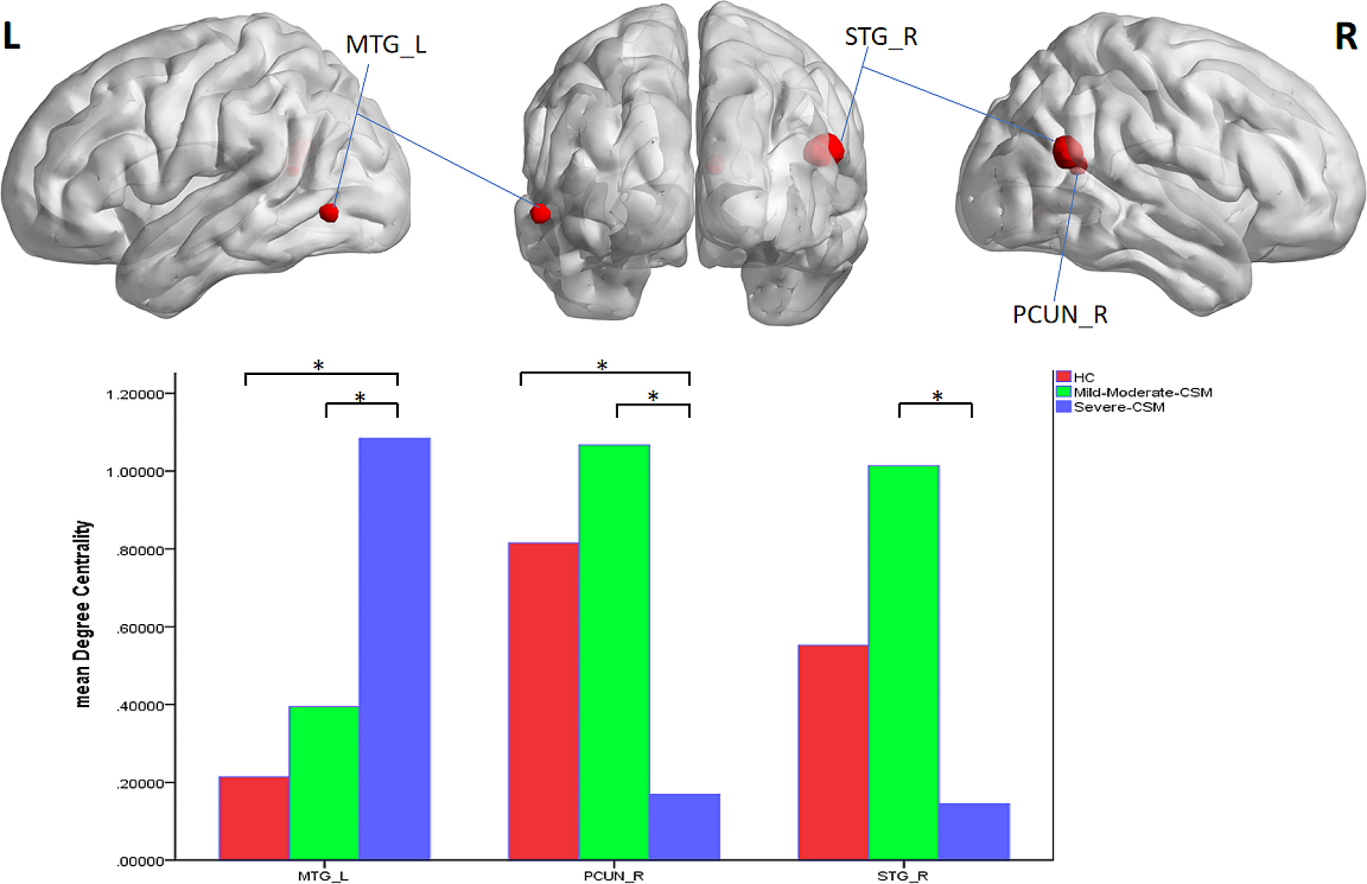
DC differences between mild-moderate, severe patients and healthy controls

The differences in DC among groups were obtained using ANCOVA with post-hoc analysis. As shown in Table 3 and Fig. 3, significant group differences in DC values primarily exist in the left middle temporal gyrus, right superior temporal gyrus and precuneus (voxel *P* < 0.001, cluster *P* < 0.05, FDR corrected). Table 4 lists the Brodmann’s Area (BA) regions, peak MNI coordinates, clusters size, and peak T-value of the differences in the DC values between the three groups. Compared with HCs, the DC values of left middle temporal gyrus exhibited a progressive increase in CSM groups, among which HCs were the lowest, significantly lower than those in mild to severe degree of CSM group (*P* < 0.05). Interestingly, the DC values of the right superior temporal gyrus and precuneus showed a decrease after increase with the severity increased. Among them, DC values in the area of precuneus in severe CSM group were significantly lower than those in mild-moderate CSM and HCs (*P* < 0.05), and the DC values of superior temporal gyrus in severe CSM group was significantly lower than that in the mild-moderate CSM group (*P* < 0.05).

**Table 3:** Differences of DC values in brain regions between mild, moderate and severe CSM groups and healthy control groups

Group	*N*	MTG_L	PCUN_R	STG_R
HC	25	0.106 ± 0.670	0.880 ± 0.662	0.538 ± 0.627
Mild-Moderate CSM	22	0.527 ± 0.233	1.009 ± 0.672	0.862 ± 0.698
Severe CSM	18	0.869 ± 0.192	0.169 ± 0.535	0.145 ± 0.598
F		11.460	9.906	6.134
P		0.000	0.000	0.004

Note MTG_L, left middle temporal gyrus; PCUN_R, right precuneus; STG_R, right superior temporal gyrus; Bonferroni correction after one-way analysis of covariance between groups (*P* < 0.05)

**Table 4:** Comparison of the DC value of different brain regions between CSM patients and healthy controls

Brain region	Brodmann area	Peak MNI coordinate			Voxels	Peak value
		X	Y	Z		
MTG_L	21	-60	-66	-6	7	14.63
PCUN_R	7	9	-48	18	7	10.18
STG_R	22	51	-54	21	24	12.60

*Note * MNI: Montreal Neurological Institute; FDR correction, voxel *P* < 0.001, cluster *P* < 0.05

### Relationship between imaging changes and clinical characteristics

**Fig. 4:**
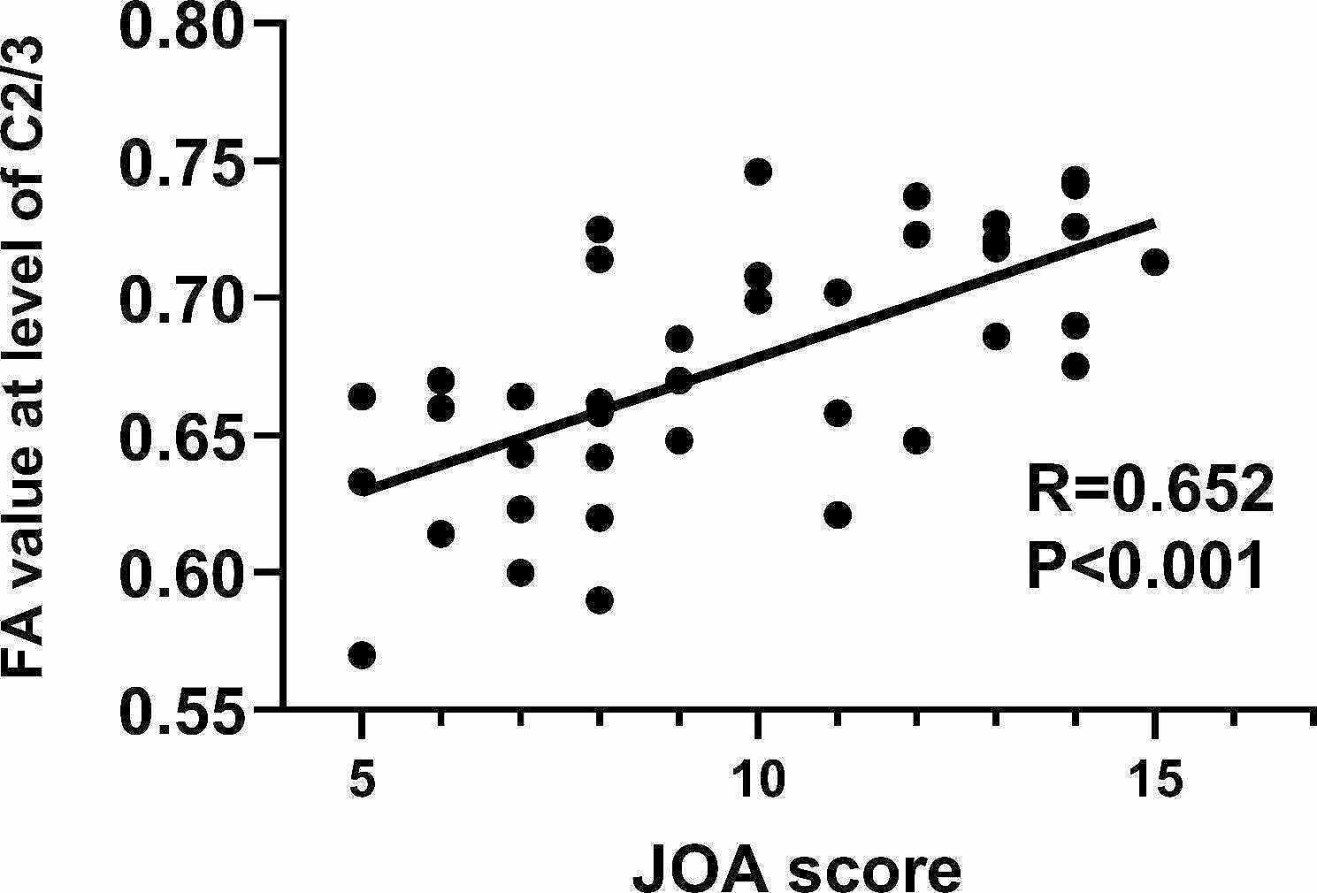
Correlation analysis between JOA score and dorsal column FA value at level of C2/3 in CSM patients

**Fig. 5:**
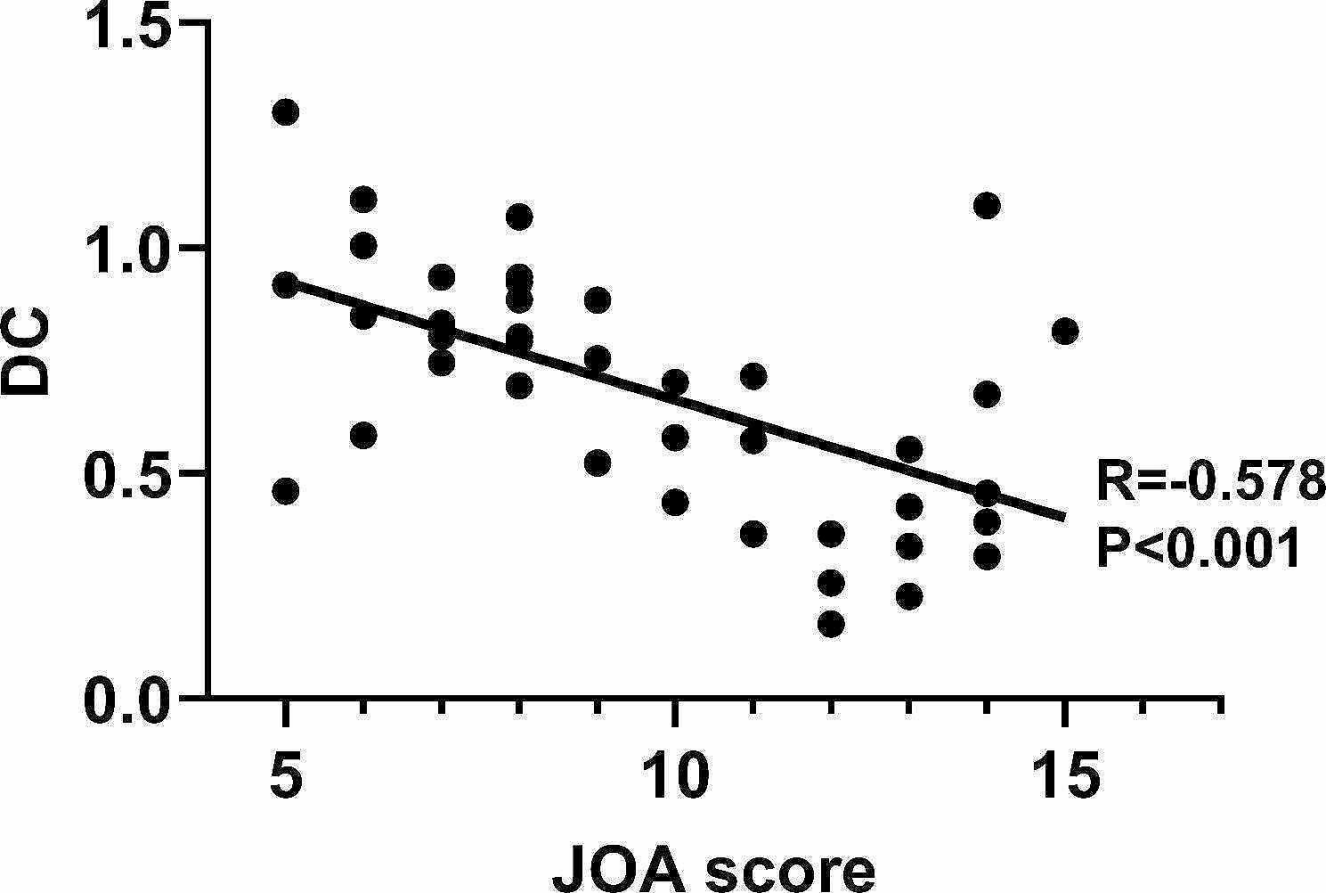
Correlation analysis between JOA score and DC value of left middle temporal gyrus

We analyzed the relationship between the clinical characteristics (JOA and NDI scores) and imaging changes in patients with CSM. Our findings indicated that the FA values of dorsal column in the level C2/3 positively correlated with the JOA(*r* = 0.652, *P* < 0.001) (Figure 4); and we also find negative correlation between their JOA and abnormal DC values of left middle temporal gyrus(*r*=-0.578, *P* < 0.001). (Fig. 5)

### Predicted the severity of CSM curve of DC and FA values

**Fig. 6:**
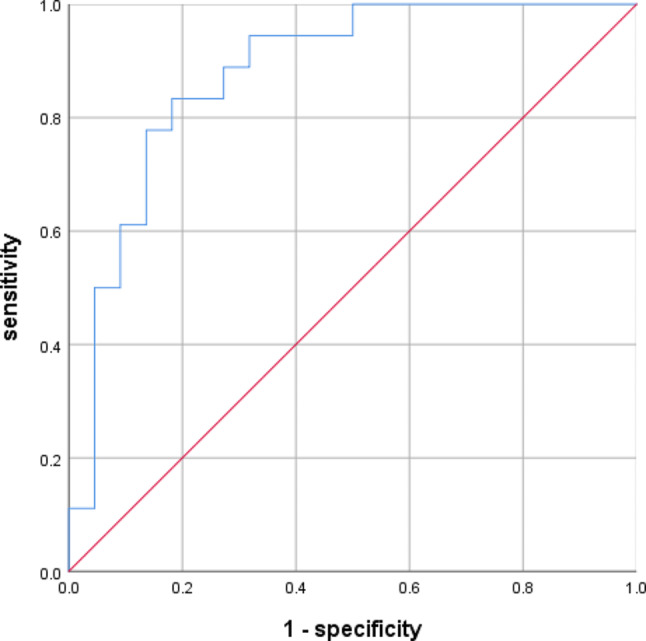
The ROC curves of the DC values for the prediction of severity of CSM

**Fig. 7:**
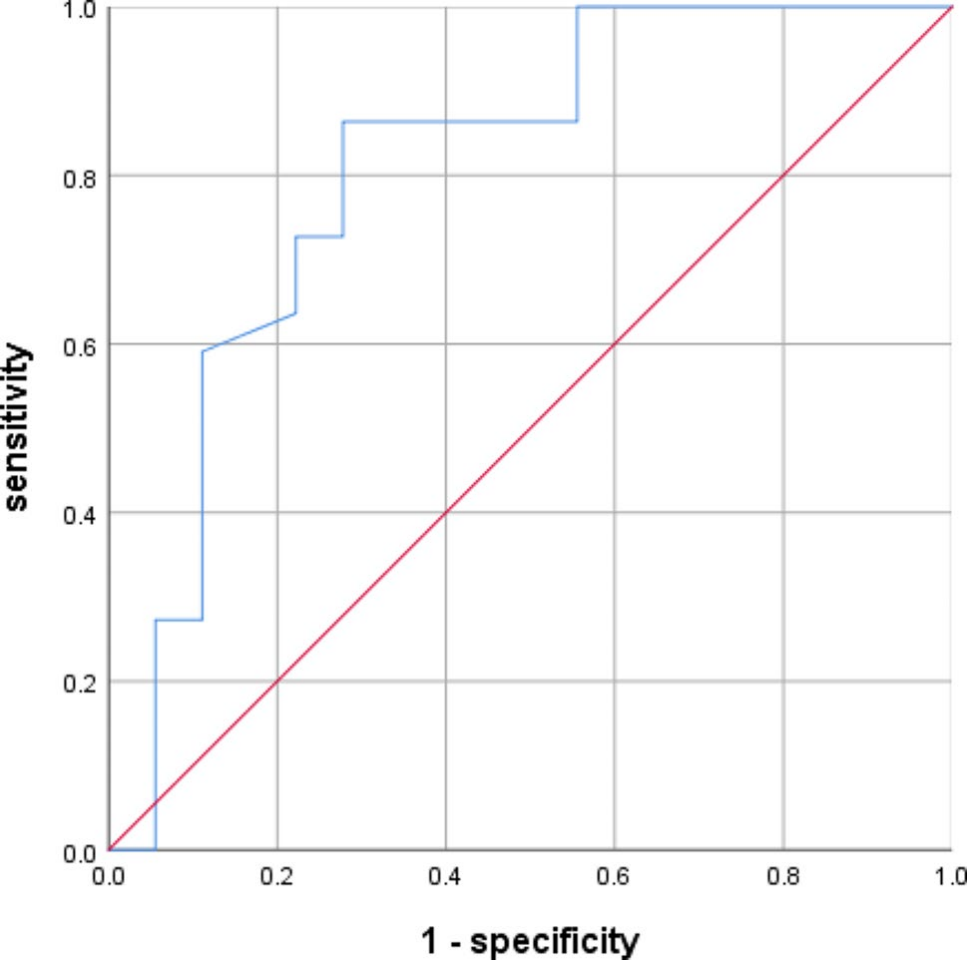
The ROC curves of the FA values for the prediction of severity of CSM

The ROC curves of DC and FA values for the prediction of severity of CSM are shown in Figs. 6 and 7. The AUC of the DC value was 0.879([95% CI, 0.771–0.987]) and the AUC of the FA value was 0.808([95% CI, 0.663–0.953]).

## Discussion

In this study, FA was used to quantify spinal cord compression severity in CSM patients using a newly developed method with high reproducibility. The association between spinal cord compression and brain function connectome was assessed using clinical characteristics. In addition, the brain function was assessed using degree centrality. The results indicate that DC abnormalities mostly in the left middle temporal gyrus, right superior temporal gyrus and precuneus and they are influenced by the degree of compression. We also found that there was negative correlation between DC values of left middle temporal gyrus and JOA, and the FA values of dorsal column in the level C2/3 positively correlated with the JOA.

Up to now, there is no unified standard for the measurement of spinal cord DTI parameters. To avoid the compressed area of the spinal cord, which could yield biased and incorrect measurements [22, 23], we have selected the level of C2/3 for ROI analysis. Jones et al. [24] reported that FA values measured at the proximal position of the spinal cord (C2/3) had the best correlation with clinical symptoms. In addition, most studies have not considered the physiological anatomy of the spinal cord, especially partial volume effects caused by the compression of the myelon. Spinal cord analysis based on SCT exhibited high reproducibility [20] and is expected to describe each anatomic structure in the spinal cord [25] more precisely than the previous ROI-based studies. Our results showed that the FA value of dorsal column in CSM patients decreased and was positively correlated with JOA, indicating that with JOA scores dropping, the severity of cervical spinal cord increased; and ascending or descending spinal cord fiber bundles were damaged and reduced, resulting in decreased FA, which was consistent with previous studies [26, 27].

Previous studies using the resting-state degree centrality analysis approach have revealed that the default-mode network (DMN) related regions are the main hubs of our human brain and have stability [28, 29]. Chronic pain has been associated with functional alterations in the DMN and differences in resting-state functional connectivity between patients and controls are typically observed within the DMN [30, 31]. Interestingly, our findings showed DC abnormalities mostly in the left middle temporal gyrus, right superior temporal gyrus and precuneus, which coincides with the DMN.

Our study showed that the DC values of the left middle temporal gyrus increased gradually in patients with CSM from mild-moderate to severe. CSM patients with more intense spinal cord injury recruit larger regions of the cortex, which suggests that this adaptation is a compensatory response to neurological injury and tissue damage in the spinal cord. We speculated that some patients initiated more cortical reorganization thus interrupting the normal cortex function leading to a higher symptom burden. However, another explanation for this phenomenon may also exist that those with severe myelopathy initiate more cortical recombination to compensate for clinical symptoms, which may be maladaptive changes that affect the sensorimotor recovery process.

Another important finding in the current study is that the DC values of the right superior temporal gyrus and precuneus in patients with CSM increased and then decreased with the severity increased. The superior temporal gyrus is regarded as important components of the emotion-regulation circuitry and morphometry of it can be a possible biomarker in schizophrenia [32],and alterations in this region may be associated with mental symptoms of CSM such as schizophrenia [33]. The precuneus is another core brain region of DMN, which has high metabolism and vulnerability. An improved structural model of the white-matter anatomy of the precuneus can demonstrate its unique cerebral connections with adjacent regions which can provide additional clarity on its role in integrating information across higher-order cerebral networks like the DMN [34]. Precuneus dysfunction is considered to be engaged in collection and evaluation of information, self-referential mental activity, and extraction of episodic memory [35]. Zhao et al [5] also found functional state changes in precuneus besides primary motor cortex in CSM patients. Decreased DC values in both the regions, based on the functional network integrity perspective, represented fewer correlated activity and impaired roles of these hubs in facilitating neural network communication [36].Therefore, we speculate that the abnormal properties of brain functional network of CSM patients at rest will change to some extent with the progress of the disease, and the neural activity tends to be maladjusted and disordered. Stam et al. [37] proposed that the main reason for this change is the overload or failure of the hub to handle information traffic in brain networks. Based on the above research background, we guess that the changes in default-mode network-related regions may be neuroimaging biomarkers for CSM patients and enable the identification of clinical targets for early treatment. However, the role of these changes in CSM disease progression and recovery is currently unknown because we didn’t conduct longitudinal studies. And lifestyle habits, smoking status, and level of physical activity, which may in fact be responsible for both effects, should take into consideration.

.

There are several limitations in the present study. First, the sample size was relatively small, which may have limited statistical efficiency. Thus, additional studies with larger sample size of CSM patients are needed to improve the power of the statistical analysis of our results. Second, CSM patients after decompressive surgery were not recruited in this study. Future longitudinal studies should include postsurgical CSM patients and follow-up for a long time to better establish a cause and effect relationship between the variables analyzed. Third, single prediction markers are easy to use in practice but lack high accuracy. Random forest, as a traditional machine learning method, has been demonstrated high performance in disease prediction. Later, we can develop a random forest model to predict the severity of CSM. Finally, this was a non-blinded experiment and therefore subject to various biases. Besides, a linear regression modeling can control covariates interference to get more scientific results.

## Conclusion

Our results preliminarily depict the distribution of core network hubs in the brain of CSM patients. Brain functional plasticity results from micro-structural damage to the cervical cord in CSM. The degree of brain function remodeling may be a quantitative imaging indicator to assess the condition of CSM patients, which requires larger samples to verify.

## Data Availability

The datasets generated during this study are available from the corresponding author upon reasonable request.

## Abbreviations

CSM: Cervical spondylotic myelopathy
HC: Healthy control
fMRI: Functional magnetic resonance imaging
JOA: Japanese Orthopedic Association Score
NID: Neck Disability Index
FA: Fractional anisotropy
DC: Degree centrality
FC: Functional connectivity
DTI: Diffusion tensor imaging
ALFF: Amplitude of low-frequency fluctuations
ROI: Regions of interest
DMN: Default-mode network
AUC: Areas under the curve
